# Protocol investigating the clinical utility of an objective measure of attention, impulsivity and activity (QbTest) for optimising medication management in children and young people with ADHD ‘QbTest Utility for Optimising Treatment in ADHD’ (QUOTA): a feasibility randomised controlled trial

**DOI:** 10.1136/bmjopen-2017-021104

**Published:** 2018-02-15

**Authors:** Charlotte L Hall, Marilyn James, Sue Brown, Jennifer L Martin, Nikki Brown, Kim Selby, Julie Clarke, Hena Vijayan, Boliang Guo, Kapil Sayal, Chris Hollis, Madeleine J Groom

**Affiliations:** 1 Institute of Mental Health, Division of Psychiatry & Applied Psychology, University of Nottingham, Nottingham, UK; 2 Division of Rehabilitation and Ageing, University of Nottingham, Nottingham, UK; 3 Department of Community Paediatrics, Medway NHS Foundation Trust, Kent, UK; 4 Grantham and District Hospital, United Lincolnshire Hospitals NHS Trust, Grantham, UK; 5 North East London NHS Foundation Trust, Acorn Centre, Romford, UK; 6 Queen’s Medical Centre, University of Nottingham, Nottingham, UK

**Keywords:** ADHD, QbTest, continous performance test (CPT), medication, titration, treatment

## Abstract

**Introduction:**

Attention-deficit hyperactivity disorder (ADHD) is characterised by symptoms of inattention, hyperactivity and impulsivity. To improve outcomes, the National Institute for Health and Care Excellence ADHD guidelines recommend regular monitoring of symptoms when children commence medication. However, research suggests that routine monitoring rarely happens, and clinicians often rely on subjective information such as reports from parents and teachers to ascertain improvement. These sources can be unreliable and difficult to obtain. The addition of an objective test of attention and activity (QbTest) may improve the objectivity, reliability and speed of clinical decision-making and so reduce the time to identify the optimal medication dose. This study aims to assess the feasibility and acceptability of a QbTest medication management protocol delivered in routine healthcare services for children with ADHD.

**Method and analysis:**

This multisite feasibility randomised controlled trial (RCT) will recruit 60 young people (aged 6–17 years old), diagnosed with ADHD, and starting stimulant medication who are seen by Child and Adolescent Mental Health Services or Community Paediatric services. Participants will be randomised into one of two arms. In the experimental arm (QbTest protocol), the participant will complete a QbTest at baseline (prior to medication initiation), and two follow-up QbTests on medication (2–4 weeks and 8–10 weeks later). In the control arm, participants will receive treatment as usual, with at least two follow-up consultations. Measures of parent-, teacher- and clinician-rated symptoms and global functioning will be completed at each time point. Health economic measures will be completed. Clinicians will record treatment decision-making. Acceptability and feasibility of the protocol will be assessed alongside outcome measure completion rates. Qualitative interviews will be conducted.

**Ethics and dissemination:**

The findings will be used to inform the development of a fully powered RCT. The results will be submitted for publication in peer-reviewed journals. The study has ethical approval.

**Trial registration number:**

NCT03368573; Pre-results.

**Strengths and limitations of this study**

- The study uses a pragmatic intervention randomised controlled trial (RCT) design conducted in routine National Health Service settings.
- Adding QbTest to routine care for medication management has not yet been attempted in the UK. In line with Medical Research Council guidelines on complex interventions, we need to first establish the feasibility of the research design.
- The protocol was co-created with a multidisciplinary team of experts, including healthcare professionals, patient and public involvement members, expert statisticians and health economists, and academics.
- If the protocol is deemed feasible and acceptable, a further fully powered RCT would be necessary to determine the health and economic impact of adding QbTest to medication management for attention-deficit hyperactivity disorder.

## Introduction

Attention-deficit hyperactivity disorder (ADHD) affects 3%–5% of children and young people under 18 years old.^1^ The core symptoms include inattention, impulsivity and hyperactivity leading to significant impairments in academic and social function and increased risk of substance misuse, unemployment, criminality and mental health problems.^2 3^ Early treatment is crucial to improve symptoms and reduce the burden on the family and wider social and healthcare systems.^4^ With the increasing rates of diagnosis of ADHD, spending on ADHD medication has increased sevenfold between 1998 and 2005,^5^ and expenditure on medication treatment costs in the UK is now estimated at £78 million per year.^5 6^ This has placed increasing financial burden on health services and highlighted the need for more efficient and cost-effective services to diagnose and treat the condition. Indeed, the National Institute for Health and Care Excellence (NICE) guidelines^1^ emphasise the importance of young people with ADHD having access to the best evidence-based care in order to fulfil their potential and prevent poor outcome. However, in practice, delivery and quality of care is variable with little consistency in diagnosis or management.^7^ Improving child and adolescent mental health services is a current government priority.^8^

NICE ADHD guidelines^1^ recommend frequent monitoring of ADHD symptoms in children and young people prescribed medication to ensure first, that the best dose of medication is reached quickly for each child and second, that the effectiveness of this dose is monitored regularly, ensuring optimal outcomes are maintained with minimal side effects. The US National Institute of Mental Health Multimodal Treatment Study of ADHD (MTA)^9^ showed that frequent symptom monitoring with careful adjustment of the dose significantly improved outcomes in ADHD. In this study, the proportion of children that experienced a clinically significant reduction in symptoms was almost 60% compared with only 25% for those not subjected to this careful monitoring procedure.^9^ While treatments for ADHD are highly efficacious in carefully managed research settings,^1^ in standard community care careful monitoring is rarely possible and the outcome of treatment may be suboptimal. Audit data within the East Midlands showed that community care for ADHD falls well below the standards for titration and monitoring set out in the MTA and NICE guidelines.^7^ Aside from delays in initiating treatment caused by diagnostic uncertainty, once on medication, children may not be reviewed sufficiently frequently for clinicians to detect non-response or partial response, or to establish the optimal dose for each child. Research has demonstrated that families are often unhappy with the length of time to attain reach an optimal dose of medication (up to 18 months), with very few participants reporting titration was achieved in the 6-week time frame advocated by NICE.^10^ These issues mean that children may not experience the full benefits of medication and this has significant negative effects on their academic, social and psychological development. A further consequence of suboptimal treatment response in routine care is poor medication adherence. In the UK, 50% of patients have stopped ADHD medication after 18 months and 80% after 3 years.^11^

Current methods to judge the effectiveness of medication rely on the clinician integrating various forms of subjective information, information such as clinical rating scales completed by parents and teachers, with their own observations. However, the information provided by these sources can be contradictory, partially completed or not returned in a timely manner leading to delays in treatment decisions. Adding more objective, computerised tests to clinical care for ADHD is one approach which has received increasing clinical and research recognition.^12^ The continuous performance test (CPT) is a computerised neuropsychological test that measures the individual’s capacity to sustain attention (vigilance) and inhibit inappropriate responses (impulsivity). Several studies have noted improvement in CPT scores in children with ADHD on stimulant medication^13–15^ indicating the potential utility of these tests to aid medication management in clinical practice. However, there is a need for further research on CPTs examining the clinical utility and cost-effectiveness using randomised controlled trials (RCTs).^16^ Furthermore, a limitation of the CPT is that it does not measure the patients’ activity levels, which is a core symptom domain of ADHD. A recent systematic review^16^ indicated that a combination of a CPT with objective direct measure of bodily activity during the test may be particularly useful as a clinical tool. One test that combines the CPT with a measure of activity is the ‘QbTest’ (Qbtech, www.qbtech.com), a commercially available measure of ADHD symptoms approved by the Food and Drug Administration (FDA) (Reference: K133382). The QbTest takes approximately 20 min to complete, during which time the child/young person is seated in front of a computer and is instructed to press a hand-held responder button each time a predesignated infrequent target stimulus appears on-screen, and to withhold the response to all other stimuli. These features of QbTest measure sustained and selective attention (target detection over 600 stimulus presentations), and impulsivity (withholding the response to a non-target). Simultaneously, an infrared camera tracks the movement of a marker attached to a headband worn during the test, to measure activity. All young people aged 6–17 years can sit the QbTest providing they do not have moderate/severe learning difficulty. The test provides a summary score relevant to each symptom domain (inattention, hyperactivity, impulsivity) with reference to a large age-stratified and gender-stratified normative database.^17^ The QbTest should not be used to reach a decision about diagnosis or medication without additional clinical information but aids decision-making by providing another source of information, reducing reliance on questionnaires.

Recent research has investigated the use of the QbTest to aid in the clinical assessment of ADHD. QbTest can help differentiate ADHD from other conditions^18–20^ and audit data suggests that QbTest can reduce the number of appointments needed to confirm a diagnosis of ADHD and result in cost savings to health services.^21^ A recent RCT with health economic analysis further investigated whether the QbTest can reduce the number of appointments needed to make a diagnostic decision on ADHD.^22^ Initial qualitative findings from this trial indicate that the QbTest is acceptable to children and families and feasible to implement in routine clinical settings.^23^ Furthermore, the qualitative interviews revealed that some clinicians currently use QbTest to: improve confidence in diagnosis before initiating medication; reassure families, young people and schools of medication efficacy to improve adherence and review medication effects at follow-up to aid decisions around dose adjustment.^23^ These findings highlight the potential clinical utility of the QbTest in medication management. In support of this, other research has shown that QbTest is sensitive to the effects of stimulant medication^24^ and, in a placebo-controlled trial of atomoxetine that performance improvements correlate with blinded observer ratings of ADHD symptoms.^25^ It has also shown some utility in identifying partial or non-responders after a single dose of methylphenidate.^26^ Another study in adults with ADHD showed that the QbTest was more sensitive to medication effects than a standardised rating scale.^27^

Although promising, few of these previous studies were conducted in the UK. Moreover, although some clinics within the UK, Europe and the USA are using QbTest to aid medication management, there is no standard approach and most clinics still rely on traditional approaches of using rating scales and clinical judgement. There is a need to formally evaluate the role of the QbTest to aid medication management in ADHD and assess whether the test should be routinely incorporated in healthcare services. In line with the Medical Research Council guidance on evaluating complex interventions (interventions (www.mrc.ac.uk/complexinterventionsguidance), the aim of this ‘QbTest Utility for Optimising Treatment in ADHD’ (QUOTA) study is to assess the feasibility and acceptability of a novel QbTest medication management protocol in a parallel group, single-blind, feasibility RCT with embedded qualitative evaluation. To ascertain the clinical utility of the protocol in standard practice, treatment as usual (TAU) was the chosen comparator. The findings from this study will be used to inform the decision to conduct a fully powered, definitive RCT investigating whether QbTest can help reduce the time to reach an optimal, effective medication dose.

## Methods and analysis

QUOTA trial commenced on 1 April 2017 and consists of two stages: stage 1 consisted of a series of three expert workshops which were conducted with the aim of designing QUOTA research protocol. Stage 2 consists of the feasibility RCT.

### Stage 1: expert workshop summary

The research study measures and medication management protocol were designed through a series of three expert workshops held from April to July 2017. The workshops consisted of up to 21 multidisciplinary experts including: 4 patient and public involvement (PPI) members (parents of young people with ADHD; including coauthor NB), 1 education expert, 2 representatives and clinical advisors from Qbtech, 1 health economics expert, 9 healthcare professionals (including consultant psychiatrists, paediatricians and nurse specialists incorporating coauthors CLH, KSa, JC, KSe), 2 academic team members (MJG and CLH) and 2 representatives from National Institute for Health Research Health Technology Assessment MindTech (JLM and SB), who also bought additional PPI expertise.

Through group discussion the expert panel made decisions on: the role and frequency of the QbTest in the medication protocol, the selection and frequency of outcome measures, the design of the health economic resource use measures and clinician proforma.

### Stage 2: feasibility RCT

#### Trial design

**Figure 1:**
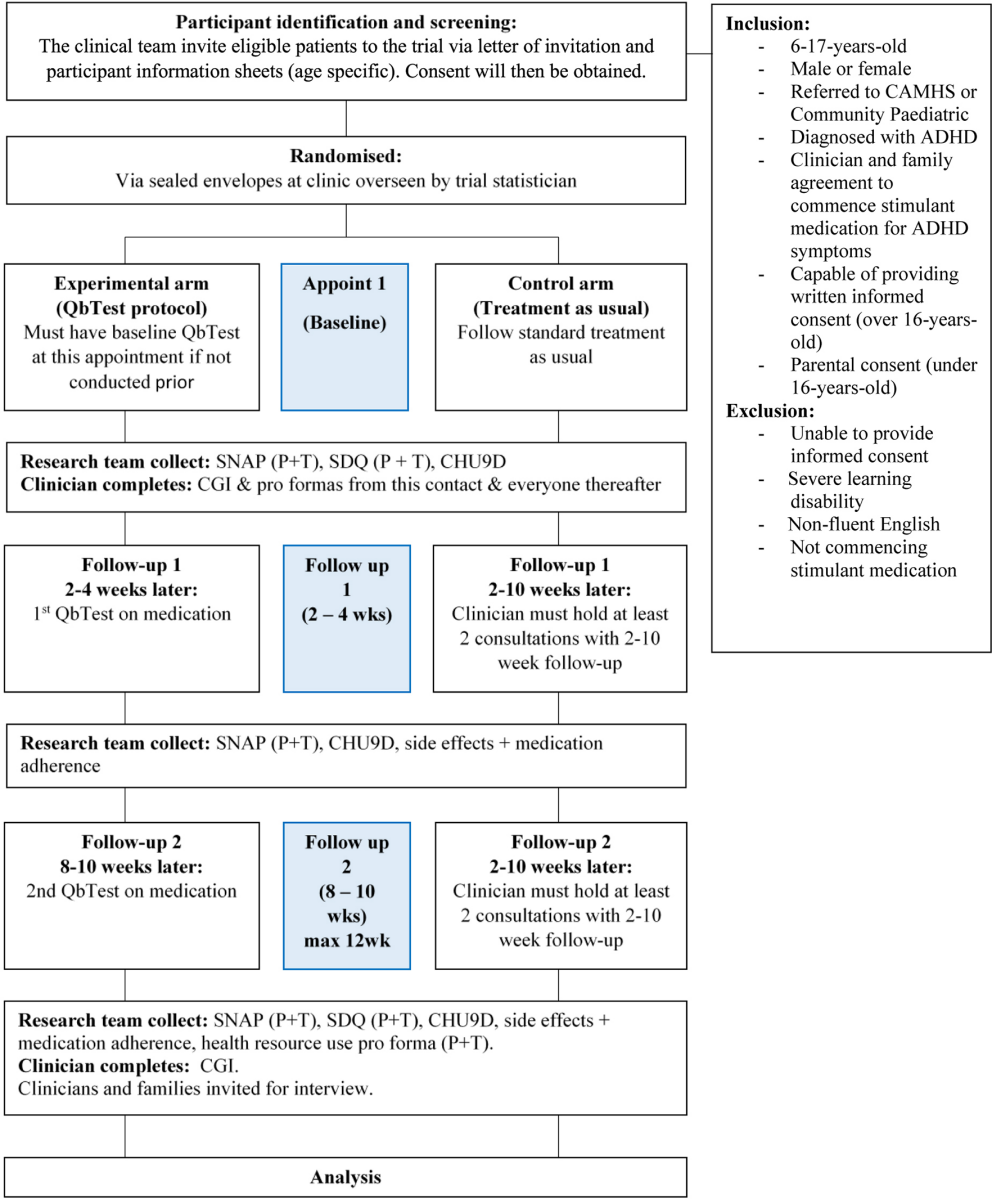
Participant flow diagram. ADHD, attention-deficit hyperactivity disorder; CAMHS, Child and Adolescent Mental Health Services; CGI, Clinical Global Impressions Scale; CHU9D, Child Health Utility; SDQ, Strengths and Difficulties Questionnaire; SNAP-IV, Swanson, Nolan and Pelham IV.

The study is a parallel group, single-blind multicentre feasibility RCT, which explores feasibility and acceptability of a QbTest medication management protocol, using quantitative, qualitative and health economic evaluations. The trial is registered with ClinicalTrials.gov (www.clinicaltrials.gov; NCT03368573) and is at the pre-results stage. The study flow is outlined in figure 1. The trial consists of two arms.

##### Experimental arm (QbTest group)

In this arm, participants will receive standard TAU plus three QbTests. The participant will complete a QbTest at baseline, prior to medication initiation (if a QbTest has not already been completed <12 weeks prior to medication initiation as part of their diagnosis), and again at follow-up 1 (2–4 weeks later) and follow-up 2 (8–10 weeks later). The clinician will use the QbTest scores to inform their clinical decision-making regarding medication decisions (ie, to inform titration, drug choice or treatment switch/termination).

##### Control arm (TAU)

In this arm, participants will receive standard TAU. However, to provide some control over the possible increased clinical contact in the experimental arm, clinicians are requested to make at least two contacts with the participant during the 12-week follow-up period. These contacts may take place over telephone.

The patients usual care team will be responsible for conducting the QbTest in clinic appointments. The QbTest will be only be conducted by trained QbTest clinicians. Although TAU differs across sites/clinicians’/cases, it typically involves clinical interviews with the parents/carer/young person to ascertain improvement in symptoms, and sometimes collection of standardised outcome measures. The TAU care practices will be recorded on a specifically created clinician-completed pro forma (see the Measures section). There are no prohibited concomitant interventions. Given this study is assessing the feasibility of the protocol no measures will be taken to improve protocol adherence.

### Setting

Child and Adolescent Mental Health Services (CAMHS) and Community Paediatric clinics across three different National Health Service (NHS) Trusts in England are participating in the trial, including Medway NHS Foundation Trust (KSe), United Lincolnshire Hospitals NHS Trust (JC), North East London NHS Foundation Trust (HV).

### Recruitment and eligibility

Recruitment is scheduled to start 1 December 2017. Patients with a confirmed ADHD diagnosis and commencing stimulant medication for ADHD will be invited to participate in the research based on the following criteria:

#### Inclusion criteria

- age 6–17 years old (at the time of consent)
- male or female
- referred to CAMHS or Community Paediatric services
- diagnosed with ADHD
- clinician and family (parent/carer and young person/child) agreement to commence stimulant medication for ADHD symptoms
- capable of providing written informed consent (over 16 years old)
- parental consent (under 16 years old).

#### Exclusion criteria

- unable to provide informed consent
- severe learning disability (to be assessed by clinical judgement)
- non-fluent English
- not commencing stimulant medication (either not started on medication at all or started on a non-stimulant medication).

Eligibility will be determined by the treating clinician who has read and approved the protocol. Written information about the trial will be provided to families by their treating clinical team at the point where a decision to start stimulant medication has been agreed. There are four types of participant information sheets: one for parents/caregivers, one for young people aged 16 years and older, one for young people aged 12–15 years old and one for children aged 6–11 years old. The information sheets were developed with our PPI group.

Clinicians will be encouraged to ask patients if they have any questions/queries before signing consent and will have sufficient knowledge of the research protocol to answer anticipated questions. Families may consent into the study at the appointment they first receive the information sheet, once they have had time to discuss the study and ask any questions with the clinician. The PPI group felt this would not put undue stress on families and was necessary to avoid any delays in medication initiation for those wishing to participate. Clinic invitations will be updated on a password-protected database, recording numbers invited, numbers declined and reasons for decline. Each site will be informed of their monthly recruitment target required in order to meet the target sample size.

### Measures

Blinded outcome assessors will be fully trained in all trial assessments and will be responsible for the delivery, monitoring, completion and data entry of all outcome measures.

Given this is a feasibility RCT, there is no specified primary outcome. The primary outcome for a future definitive RCT will be selected through our workshops (stage 1) and postintervention interviews (stage 2) as being most clinically meaningful and important to our workshop experts, and also is shown to be acceptable for participants to complete in the feasibility RCT. The measures collected during this trial include:

- Swanson, Nolan and Pelham version IV (SNAP-IV)^28^: SNAP-IV is a short, 26-item questionnaire designed to assess ADHD symptoms. SNAP-IV will be completed by parents/carers and teachers at baseline, follow-up 1 and follow-up 2. A 25% reduction in scores from baseline to follow-up 2 was identified in the workshops (stage 1) as an appropriate potential primary outcome measure.
- Strengths and Difficulties Questionnaire (SDQ)^29^: The SDQ is a brief, 25-item behavioural screening questionnaire which can be used as part of a clinical assessment for ADHD. The questionnaire also contains a brief impact supplement which assesses the burden and impact of symptoms.^30^ The SDQ will be completed by parents/carers and teachers at baseline and follow-up 2.
- Clinical Global Impressions Scale (CGI)^31^: The CGI is a clinician-completed measure designed to measure the clinician view of global functioning prior to, and after, treatment initiation. The questionnaire consists of two items, one measuring symptom severity and one measuring change since treatment. The CGI will be completed by the clinician at baseline and follow-up 2.
- Child Health Utility (CHU9D)^32^: The CHU9D is a quality of life measure designed for the economic evaluation of interventions for young people. The CHU9D will be completed by parents/carers/young people at baseline, follow-up 1 and follow-up 2.
- QbTest: Q scores for attention, impulsivity and activity will be compared between the two groups. The QbTest is completed by the young person at baseline (if not conducted as part of the diagnostic assessment within 12 weeks of medication initiation), follow-up 1 and follow-up 2.
- Side effects scale.^33^ A side effects scale will be completed by parents/carers/young people and teachers at follow-up 1 and follow-up 2 to check for any differences in side effects between the two treatment groups.
- Medication adherence questionnaire: To ascertain that participants have been taking medication, they will be asked to complete a brief questionnaire created specifically for this study which asks how often they have taken their medication over the past 4 weeks. The questionnaire will be completed by parents/carers/young people at follow-up 1 and follow-up 2.
- Resource Use—Services for Health (RUSH) and Resource Use—Services in Education (RUSE): To collect relevant health economic information, two-tailored resource use tools will be used to measure the use of services used by the family and to ascertain indirect costs (such as time off work). More specifically, RUSE measures the use of additionally education resources. The measures were based on the Client Service Receipt Inventory,^34^ and refined for use in this study by our multidisciplinary group of members (including PPI, clinicians and a health economic expert (MJ)) in our expert workshops (stage 1). The measures will be completed by parents (RUSH) and teachers (RUSE) at follow-up 2.
- Clinical pro forma: As part of the expert workshops, a specifically created pro forma was designed for completion by clinicians after each consultation with the young person and/or family. The pro forma documents information about appointment duration, diagnosis and changes to medication/treatment. The pro forma can be provided by contacting the corresponding author.

SNAP-IV,^35^ SDQ,^36^ CGI,^37^ CHU9D^38^ and side effects scale^33^ have established reliability, validity and history of use in clinical and research settings. As this is a feasibility study, there are no plans to promote retention and follow-up measure completion, but completion rates will be recorded to inform the future RCT.

Additionally, a subsample of 10–15 participants (parents/carers and/or young people) in the experimental arm will be interviewed about their experiences of the trial, including their opinion on randomisation; within this subsample, we will include participants who did not complete the trial, if possible, acknowledging that the views and experiences of non-completers may also provide useful insight into acceptability. A subsample of 10 in the control arm will be interviewed about their experiences of ADHD medication. The subsample will be chosen at random from each participating site, using a random number generator. All clinicians participating in the feasibility RCT will be interviewed, and asked to comment on any local factors that influenced delivery of the protocol at their site, providing early insight into factors that might influence delivery of the multisite RCT and future implementation of the protocol into the NHS. All interviews will take place after their duration in the RCT has been completed to avoid any impact on outcome measures. Interviews will be digitally recorded, transcribed and analysed. The results of these interviews will be used to inform any refinement of the protocol to improve its acceptability before embarking on the definitive RCT.

Table 1 displays the study measures, the informant and the time point of completion. All measures will have a 1-month window for completion, with the exception of the clinic pro forma which must be just after the clinic appointment and the QbTest which will be completed within the specified time frame. For participants who withdraw from the trial, the outcome measures already collected will be included in analysis, and no further outcome measures will be collected.

**Table 1:** Table of study measures

Measure (and informant)	Baseline	Follow-up 1 (2–4 weeks)	Follow-up 2 (8–10 weeks)
SNAP-IV (P&T)	x	x	x
SDQ (P&T)	x		x
CGI (C)	x		x
CHU9D (P&YP)	x	x	x
Medication adherence (P&YP)		x	x
Side effects (P&YP)		x	x
QbTest (YP)*	x	x	x
RUSH (P)			x
RUSE (T)			x
Pro forma (C)†	x	x	x
Subsample for interview (P, YP, C)			x

*Experimental arm only.

†Pro forma completed at every appointment.

C, clinician; CGI, Clinical Global Impression Scale; CHU9D, Child Health Utility; P, parent/carer; RUSE, Resource Use—Services in Education; RUSH, Resource Use—Services for Health; SDQ, Strengths and Difficulties Questionnaire; SNAP-IV, Swanson, Nolan and Pelham 4th edition; T, teacher completed; YP, young person.

### Sample size and justification

The required sample is 60 participants, 30 per study arm. Participants will be families (parents/carers and children/young people) whose child/young person is about to commence stimulant medication for ADHD. These sample sizes are large enough to test the feasibility of the research procedures and to establish a mean and SD on each outcome measure (Hertzog; 2008). The study has an 8-month recruitment period, requiring 7.5 participants to be recruited into the study each month. Based on findings from the AQUA-Trial (which included the three sites used in this trial) this target is achievable. Recruitment rates and the final target will be used to inform the decision to proceed to a definitive RCT.

### Randomisation and blinding

After obtaining informed consent, participants will be randomised on a 1:1 ratio into either the QbTest medication arm (experimental arm) or TAU (control arm). Randomisation will take place via sealed opaque envelopes generated by our study statistician (BG). The sealed envelopes will be provided to the clinic sites and opened at the point of consent by the clinician.

All participants will undergo the same research measures, with the exception of the QbTest, which will only be in the experimental arm. Outcome assessors for all measures will be blind to which arm the participant is in. There are no anticipated events in which participant unblinding would be necessary.

### Data analysis plan

As a feasibility study, data analysis will be mainly descriptive, as recommended by Lancaster^39^ and Lancaster *et al*.^40^ All measures will be summarised by group across follow-up time with mean (SD) for normally distributed data, median (IQR) for skewed variable and frequency (percentage) for categorical measures. Together with site-level intraclass correlation coefficient, treatment effects and 95% CI will be derived using multilevel modelling. Recruitment rate and retention rate will also be calculated from the data. This information will be used to inform the future definitive RCT design. All statistical analysis will be conducted using STATA V.15. No interim analysis is planned.

To inform the acceptability and feasibility of the study design the following indices will be recorded and analysed: (1) *acceptability of randomisation*—record of the number of patients who do not participate stating randomisation as the reason for non-participation, drop-out rates of randomisation, errors in randomisation per site; (2) *acceptability of study design*—record of the number of eligible participants at each site and the percentage that consent to take part in the study, number of withdrawals at each follow-up time point; (3) *acceptability of outcome measures*—record of completion rates for outcome measures, percentage of data collected online, via telephone or postal completion; (4) *acceptability/feasibility of the protocol*—record of non-adherence of healthcare professionals to the protocol. These reasons will be further explored in the qualitative interviews and (5) *feasibility of a future definitive RCT*—record an estimate of the hours per week spent conducting the RCT and estimate the number of researchers required and the time commitment for healthcare professionals in a future RCT.

The qualitative interviews will shed light on contextual and other factors that might affect implementation of QbTest (both as part of the trial, and within the broader processes of care) and will be used to refine the RCT design (and QbTest implementation more broadly) if appropriate. The qualitative interviews will be transcribed verbatim and analysed thematically following the guidelines of Braun and Clarke.^41^ The quantitative and qualitative findings will be used to determine the feasibility and acceptability of the medication protocol and research study design, and inform the decision to proceed to a fully powered RCT.

### Monitoring

#### Management and oversight

Recruitment and study progress will be overseen by our project management group (PMG), which includes all site principal investigators (JC, KSe, HV), a PPI representative (NB), the chief investigator (MJG), the trial manager (CLH) and the study team (CH, SB, MJ, BG, KSa). The PMG will meet every 6 months, however, any severe slippages in recruitment or study milestones will be reported to the group immediately by the trial manager (CLH). Given this is a feasibility study, a data monitoring committee is not necessary.

#### Adverse events

All adverse events that occur will be assessed for seriousness, expectedness and causality. The chief investigator (MJG) and the medical expert (CH) shall be informed immediately of any serious adverse events and shall determine seriousness and causality in conjunction with any treating medical practitioners. All treatment-related serious adverse events will be recorded and reported to the Research Ethics Committee (REC). There are no anticipated adverse events arising from this study.

#### Audit

The Trial Coordinator or a nominated designee of the Sponsor shall carry out monitoring of trial data as an ongoing activity. A sample (10%) of case report forms will be checked on a regular basis for verification of all entries made. Where corrections are required these will carry a full audit trail and justification. Trial data and evidence of monitoring and systems audits will be made available for inspection by the REC as required.

## Ethics and dissemination

Health Research Authority approvals have been granted from the three participating trusts. Only the research team will have access to the study data, which will be stored in secure locked files or password-protected databases. Data will be available for inspection by the ethics committee on request. Changes to the protocol will be communicated to the ethics committee and trial registries by the trial manager (CLH). The process for obtaining participant informed consent or assent and parent/guardian/ teacher informed consent will be in accordance with the ethical guidance and Good Clinical Practice. The investigator or their nominee and the participant or other legally authorised representative (such as the child’s parent) shall both sign and date the informed consent forms (online supplementary appendix A, B) before the person can participate in the study. Where the young person is 16 years and over, written consent will be required from the young person and parent alike. Where the young person is under 16 years, written parental consent will be required, alongside the young person’s written or verbal assent. Teachers will also be asked to sign a consent form (online supplementary appendix C), if teachers do not sign consent the participant is still eligible for the study but no teacher measures will be collected. Individual participant medical information obtained as a result of this study are considered confidential and disclosure to third parties is prohibited unless warranted by an adverse event. Participant confidentiality will be further ensured by using identification code numbers to correspond to treatment data in the computer files. No post-trial care is required.

10.1136/bmjopen-2017-021104.supp3 Supplementary file 3

The findings from the trial will be used to inform the design, feasibility and acceptability of a future, fully powered RCT. The findings will be published in peer-reviewed journals, presented at relevant conferences and disseminated to the public via lay summaries co-created with our PPI group. All outputs will be authored by the research team and will not involve professional writers. Access to the full protocol and statistical codes are available on request to the corresponding author.

## Trial sponsor

Nottinghamshire Healthcare NHS Foundation Trust; Shirley Mitchell. Duncan Macmillan House, Porchester Road, Mapperley, Nottingham, UK, NG3 6AA Shirley.mitchell@nottshc.nhs.uk (Reference: Groom050917).

10.1136/bmjopen-2017-021104.supp1 Supplementary file 1

10.1136/bmjopen-2017-021104.supp2 Supplementary file 2

## Supplementary Material

Reviewer comments

Author's manuscript
