# Supplementary material for: Protocol investigating the clinical utility of an objective measure of attention, impulsivity and activity (QbTest) for optimising medication management in children and young people with ADHD ‘QbTest Utility for Optimising Treatment in ADHD’ (QUOTA): a feasibility randomised controlled trial
Source: BMJ Open. 2018 Feb 15;8(2):e021104. doi: 10.1136/bmjopen-2017-021104 (PMC5829926; doi:10.1136/bmjopen-2017-021104)
Supplement: Supplementary file 3 [file bmjopen-2017-021104supp003.pdf]

Appendix C INSERT TRUST LOGO

V1.0: 10.10.2017

Study ID: IRAS 219538 **Participant Identification Number:**

Name of participant (child):

CONSENT FORM

Title of Project: QbTest Utility for Optimising Treatment in ADHD (QUOTA)

Name of Researcher: **CI: Dr Maddie Groom.**

Please initial all boxes

1. I confirm that I have read and understand the information sheet dated 10th Oct 2017 version 1.1 for the above study. I have had the opportunity to consider the information, ask questions (by email or phone) and have had these answered satisfactorily (if appropriate). ☐

2. I understand that my participation is voluntary and that I am free to withdraw at any time without giving any reason, without the child's medical care or legal rights being affected. ☐

3. I understand that all data will be kept in accordance with the Data Protection Act (1998) and that no material which could identify individual children, teachers or the school will be used in any reports of this project. ☐

4. I agree to complete questionnaires about the child's behaviour at school and provide a Provision Map if available. ☐

5. I agree to take part in the above study. ☐

Your name (PLEAST PRINT)	Date	Signature

Your role e.g. class teacher, form teacher, SENCo

To be completed by the research team:

Name of person taking consent	Date	Signature
