# Supplementary material for: Protocol investigating the clinical utility of an objective measure of attention, impulsivity and activity (QbTest) for optimising medication management in children and young people with ADHD ‘QbTest Utility for Optimising Treatment in ADHD’ (QUOTA): a feasibility randomised controlled trial
Source: BMJ Open. 2018 Feb 15;8(2):e021104. doi: 10.1136/bmjopen-2017-021104 (PMC5829926; doi:10.1136/bmjopen-2017-021104)
Supplement: Supplementary file 2 [file bmjopen-2017-021104supp002.pdf]

Appendix B INSERT TRUST LOGO

V1.0: 30.08.2017

Study ID: IRAS 219538 **Participant Identification Number:**

Name of participant (young person):

CONSENT FORM

Title of Project: QbTest Utility for Optimising Treatment in ADHD (QUOTA)

Name of Researcher: **CI: Dr Maddie Groom.**

Please initial all boxes

1. I confirm that I have read and understand the information sheet dated 10th Oct 2017 version 1.1 for the above study. I have had the opportunity to consider the information, ask questions and have had these answered satisfactorily. ☐

2. I understand that my participation is voluntary and that I am free to withdraw at any time without giving any reason, without my medical care or legal rights being affected. ☐

3. I understand that relevant sections of my medical notes and data collected during the study, may be looked at by individuals from the University of Nottingham, from regulatory authorities or from the NHS Trust, where it is relevant to my taking part in this research. I give permission for these individuals to have access to my records. ☐

4. I agree to my teacher and head teacher to be contacted and be asked to complete questionnaires about my behaviour at school and provide a Provision Map if avalaible. ☐

5. I understand if I take part in any research interviews, they will be recorded and that anoymous direct quotes from the interviews may be used in study reports. ☐

6. I agree to take part in the above study. ☐

Name of participant	Date	Signature
Name of person taking consent	Date	Signature
