# Supplementary material for: Protocol investigating the clinical utility of an objective measure of attention, impulsivity and activity (QbTest) for optimising medication management in children and young people with ADHD ‘QbTest Utility for Optimising Treatment in ADHD’ (QUOTA): a feasibility randomised controlled trial
Source: BMJ Open. 2018 Feb 15;8(2):e021104. doi: 10.1136/bmjopen-2017-021104 (PMC5829926; doi:10.1136/bmjopen-2017-021104)
Supplement: Author's manuscript [file bmjopen-2017-021104.draft_revisions.pdf]

BMJ Open is committed to open peer review. As part of this commitment we make the peer review history of every article we publish publicly available.

When an article is published we post the peer reviewers' comments and the authors' responses online. We also post the versions of the paper that were used during peer review. These are the versions that the peer review comments apply to.

The versions of the paper that follow are the versions that were submitted during the peer review process. They are not the versions of record or the final published versions. They should not be cited or distributed as the published version of this manuscript.

BMJ Open is an open access journal and the full, final, typeset and author-corrected version of record of the manuscript is available on our site with no access controls, subscription charges or pay-per-view fees (http://bmjopen.bmj.com).

If you have any questions on BMJ Open's open peer review process please email editorial.bmjopen@bmj.com

BMJ Open

Protocol investigating the clinical utility of an objective measure of attention, impulsivity and activity (QbTest) for optimising medication management in children and young people with ADHD 'QbTest Utility for Optimising Treatment in ADHD' (QUOTA): a feasibility randomised controlled trial

Journal:	*BMJ Open*
Manuscript ID	bmjopen-2017-021104
Article Type:	Protocol
Date Submitted by the Author:	11-Dec-2017

Complete List of Authors:	Hall, Charlotte; University of Nottingham, Institute of Mental Health, Department of Psychiatry and Applied Psychology<br>James, Marilyn; University of Nottingham,<br>Brown, Sue; University of Nottingham, Institute of Mental Health, Department of Psychiatry and Applied Psychology<br>Martin, Jen; University of Nottingham, Institute of Mental Health, Department of Psychiatry and Applied Psychology<br>Brown, Nikki; University of Nottingham, Institute of Mental Health, Department of Psychiatry and Applied Psychology<br>Selby, Kim; Medway NHS Foundation Trust<br>Clarke, Julie; United Lincolnshire Hospitals NHS Trust<br>Vijayan, Hena; North East London NHS Foundation Trust<br>Guo, Boliang; Institute of Mental Health, Psychiatry<br>Sayal, Kapil; University of Nottingham,<br>Hollis, Chris; University of Nottingham, Division of Psychiatry<br>Groom, Maddie; University of Nottingham

Keywords:	ADHD, QbTest, Continous Performance Test (CPT), Medication, Titration, Treatment

SCHOLARONE™
Manuscripts

Protocol investigating the clinical utility of an objective measure of attention, impulsivity and activity (QbTest) for optimising medication management in children and young people with ADHD 'QbTest Utility for Optimising Treatment in ADHD' (QUOTA): a feasibility randomised controlled trial

Authors

[1]Charlotte L. Hall, Senior Research Fellow, Institute of Mental Health, University of Nottingham, Nottingham, UK

[2]Marilyn James, Professor of Health Economics, Division of Rehabilitation and Ageing, University of Nottingham, Nottingham, UK

[3]Sue Brown, Research Fellow, NIHR MindTech, Institute of Mental Health, University of Nottingham, Nottingham, UK

[4]Jennifer L Martin, NIHR MindTech Programme Manager, Institute of Mental Health, University of Nottingham, Nottingham, UKs

[5]Nikki Brown, Patient and Public Involvement Lead, Institute of Mental Health, University of Nottingham, Nottingham, UK

[6]Kim Selby, Associate Specialist in Community Paediatrics, Medway NHS Foundation Trust, Department of Community Paediatrics, Kent, UK

[7]Julie Clarke, Locum Consultant Community Paediatrician, United Lincolnshire Hospitals NHS Trust, Grantham and District Hospital, Grantham, Lincolnshire, UK

[8]Hena Vijayan, Consultant child and adolescent psychiatrist North East London NHS Foundation Trust, Acorn Centre, Romford, Essex, UK

[9]Boliang Guo, Medical Statistician, Institute of Mental Health, University of Nottingham, Nottingham, UK

[10]Kapil Sayal, Professor of Child & Adolescent Psychiatry, Developmental Psychiatry, University of Nottingham, Queens Medical Centre, Nottingham, UK

[11]Chris Hollis, Professor of Child & Adolescent Psychiatry, Developmental Psychiatry, University of Nottingham, Queens Medical Centre, Nottingham, UK

[12]Madeleine J. Groom, Assistant Professor in in Applied Developmental Cognitive Neuroscience, Institute of Mental Health, University of Nottingham, Nottingham, UK

Corresponding Author

Charlotte Hall (*PhD)*
Institute of Mental Health, University of Nottingham, Triumph Road, Nottingham, NG7 2TU
Email: charlotte.hall@nottingham.ac.uk Tel: 0115 8232438

Key words: ADHD, Continuous Performance Test (CPT), QbTest, medication, titration, treatment
Word count: 4246 (excluding title page, abstract, summary, references and figures).

For peer review only - http://bmjopen.bmj.com/site/about/guidelines.xhtml

ABSTRACT

Introduction

Attention Deficit Hyperactivity Disorder (ADHD) is characterised by symptoms of inattention, hyperactivity and impulsivity. To improve outcomes, the National Institute for Health and Care Excellence (NICE) ADHD guidelines recommend regular monitoring of symptoms when children commence medication. However, research suggests that routine monitoring rarely happens, and clinicians often rely on subjective information such as reports from parents and teachers to ascertain improvement. These sources can be unreliable and difficult to obtain. The addition of an objective test of attention and activity (QbTest) may improve the objectivity, reliability and speed of clinical decision-making and so reduce the time to identify the optimal medication dose. This study aims to assess the feasibility and acceptability of a QbTest medication management protocol delivered in routine healthcare services for children with ADHD.

Method and analysis

This multi-site feasibility randomised controlled trial (RCT) will recruit 60 young people (aged 6-17 years-old), diagnosed with ADHD, and starting stimulant medication who are seen by Child and Adolescent Mental Health Services or Community Paediatric services. Participants will be randomised into one of two arms. In the experimental arm (QbTest protocol), the participant will complete a QbTest at baseline (prior to medication initiation), and two follow-up QbTests on medication (2-4 weeks and 8-10 weeks later). In the control arm, participants will receive treatment-as-usual, with at least two follow-up consultations. Measures of parent, teacher and clinician-rated symptoms and global functioning will be completed at each time-point. Health economic measures will be completed. Clinicians will record treatment decision-making. Acceptability and feasibility of the protocol will be assessed alongside outcome measure completion rates. Qualitative interviews will be conducted.

For peer review only - http://bmjopen.bmj.com/site/about/guidelines.xhtml

Ethics and dissemination

The findings will be used to inform the development of a fully-powered RCT. The results will be submitted for publication in peer-reviewed journals. The study has ethical approval.

Trial registration: NCT03368573

STRENGTHS AND LIMITATIONS OF THIS STUDY

- The study utilises a pragmatic intervention RCT design conducted in routine NHS settings.
- Adding QbTest to routine care for medication management has not yet been attempted in the UK. In line with MRC guidelines on complex interventions we need to first establish the feasibility of the research design.
- The protocol was co-created with a multidisciplinary team of experts including, healthcare professionals, patient and public involvement members, expert statisticians and health economists, and academics.
- If the protocol is deemed feasible and acceptable a further fully-powered RCT would be necessary to determine the health and economic impact of adding QbTest to medication management for ADHD.

INTRODUCTION

Attention Deficit/Hyperactivity Disorder (ADHD) affects 3-5% of children and young people under 18-years old[1]. The core symptoms include inattention, impulsivity and hyperactivity leading to significant impairments in academic and social function and increased risk of substance misuse, unemployment, criminality and mental health problems[2 3]. Early treatment is crucial to improve symptoms and reduce the burden on the family and wider social and healthcare systems[4]. With the increasing rates of diagnosis of ADHD, spending on ADHD

For peer review only - http://bmjopen.bmj.com/site/about/guidelines.xhtml

medication has increased seven-fold between 1998 and 2005[5], and expenditure on medication treatment costs in the UK is now estimated at £78 million per year[5 6]. This has placed increasing financial burden on health services and highlighted the need for more efficient and cost-effective services to diagnose and treat the condition. Indeed, the National Institute for Health and Care Excellence (NICE) guidelines[1] emphasise the importance of young people with ADHD having access to the best evidence-based care in order to fulfil their potential and prevent poor outcome. However, in practice, delivery and quality of care is variable with little consistency in diagnosis or management[7]. Improving child and adolescent mental health services is a current government priority[8].

NICE ADHD guidelines[1] recommend frequent monitoring of ADHD symptoms in children and young people prescribed medication to ensure firstly, that the best dose of medication is reached quickly for each child and secondly, that the effectiveness of this dose is monitored regularly, ensuring optimal outcomes are maintained with minimal side effects. The U.S. National Institute of Mental Health Multimodal Treatment study of ADHD (MTA)[9] showed that frequent symptom monitoring with careful adjustment of the dose significantly improved outcomes in ADHD. In this study, the proportion of children that experienced a clinically significant reduction in symptoms was almost 60% compared with only 25% for those not subjected to this careful monitoring procedure[9]. Whilst treatments for ADHD are highly efficacious in carefully managed research settings[1], in standard community care careful monitoring is rarely possible and the outcome of treatment may be sub-optimal. Audit data within the East Midlands showed that community care for ADHD falls well below the standards for titration and monitoring set out in the MTA and NICE guidelines[7]. Aside from delays in initiating treatment caused by diagnostic uncertainty, once on medication, children may not be reviewed sufficiently frequently for clinicians to detect non-or partial-response, or to establish the optimal dose for each child. Research has demonstrated that families are often

For peer review only - http://bmjopen.bmj.com/site/about/guidelines.xhtml

unhappy with the length of time to attain reach an optimal dose of medication (up to 18-months), with very few participants reporting titration was achieved in the six-week time frame advocated by NICE[10]. These issues mean that children may not experience the full benefits of medication and this has significant negative effects on their academic, social and psychological development. A further consequence of sub-optimal treatment response in routine care is poor medication adherence. In the U.K., 50% of patients have stopped ADHD medication after 18 months and 80% after 3 years[11].

Current methods to judge the effectiveness of medication rely on the clinician integrating various forms of subjective information, information such as clinical rating scales completed by parents and teachers, with their own observations. However, the information provided by these sources can be contradictory, partially completed, or not returned in a timely manner leading to delays in treatment decisions. Adding more objective, computerised tests to clinical care for ADHD is one approach which has received increasing clinical and research recognition[12]. The continuous performance test (CPT) is a computerised neuropsychological test that measures the individual's capacity to sustain attention (vigilance) and inhibit inappropriate responses (impulsivity). Several studies have noted improvement in CPT scores in children with ADHD on stimulant medication[13-15] indicating the potential utility of these tests to aid medication management in clinical practice. However, there is a need for further research on CPTs examining the clinical utility and cost effectiveness using randomised control trials (RCTs) [16]. Furthermore, a limitation of the CPT is that it doesn't measure the patients' activity levels, which is a core symptom domain of ADHD. A recent systematic review [16] indicated that a combination of a CPT with objective direct measure of bodily activity during the test, may be particularly useful as a clinical tool. One test that combines the CPT with a measure of activity is the 'QbTest' (Qbtech Ltd, www.qbtech.com), a commercially available measure of ADHD symptoms approved by the FDA (Ref: K133382).

For peer review only - http://bmjopen.bmj.com/site/about/guidelines.xhtml

The QbTest takes approximately 20-minutes to complete, during which time the child/young person is seated in front of a computer and is instructed to press a hand-held responder button each time a pre-designated infrequent target stimulus appears on-screen, and to withhold the response to all other stimuli. These features of QbTest measure sustained and selective attention (target detection over 600 stimulus presentations), and impulsivity (withholding the response to a non-target). Simultaneously, an infra-red camera tracks the movement of a marker attached to a headband worn during the test, to measure activity. All young people aged 6-17 years can sit the QbTest providing they do not have moderate/severe learning difficulty. The test provides a summary score relevant to each symptom domain (inattention, hyperactivity, impulsivity) with reference to a large age- and gender-stratified normative database[17]. The QbTest should not be used to reach a decision about diagnosis or medication without additional clinical information but aids decision-making by providing another source of information, reducing reliance on questionnaires.

Recent research has investigated the use of the QbTest to aid in the clinical assessment of ADHD. QbTest can help differentiate ADHD from other conditions[18-20] and audit data suggests that QbTest can reduce the number of appointments needed to confirm a diagnosis of ADHD and result in cost-savings to health services[21]. A recent RCT with health economic analysis further investigated whether the QbTest can reduce the number of appointments needed to make a diagnostic decision on ADHD[22]. Initial qualitative findings from this trial indicate that the QbTest is acceptable to children and families and feasible to implement in routine clinical settings[23]. Furthermore, the qualitative interviews revealed that some clinicians currently use QbTest to: improve confidence in diagnosis before initiating medication; reassure families, young people and schools of medication efficacy to improve adherence and review medication effects at follow-up to aid decisions around dose adjustment[23]. These findings highlight the potential clinical utility of the QbTest in

For peer review only - http://bmjopen.bmj.com/site/about/guidelines.xhtml

medication management. In support of this, other research has shown that QbTest is sensitive to the effects of stimulant medication[24] and, in a placebo-controlled trial of atomoxetine that performance improvements correlate with blinded observer ratings of ADHD symptoms[25]. It has also shown some utility in identifying partial or non-responders after a single dose of methylphenidate[26]. Another study in adults with ADHD showed that the QbTest was more sensitive to medication effects than a standardised rating scale[27].

Although promising, few of these previous studies were conducted in the UK. Moreover, although some clinics within the UK, Europe and the USA are using QbTest to aid medication management, there is no standard approach and most clinics still rely on traditional approaches of using rating scales and clinical judgement. There is a need to formally evaluate the role of the QbTest to aid medication management in ADHD and assess whether the test should be routinely incorporated in healthcare services. In line with the MRC guidance on evaluating complex interventions (interventions

(www.mrc.ac.uk/complexinterventionsguidance), the aim of this 'QbTest Utility for Optimising Treatment in ADHD' (QUOTA) study is to assess the feasibility and acceptability of a novel QbTest medication management protocol in a parallel group, single-blind, feasibility RCT with embedded qualitative evaluation. To ascertain the clinical utility of the protocol in standard practice, treatment-as-usual was the chosen comparator. The findings from this study will be used to inform the decision to conduct a fully-powered, definitive RCT investigating whether QbTest can help reduce the time to reach an optimal, effective medication dose.

For peer review only - http://bmjopen.bmj.com/site/about/guidelines.xhtml

METHODS AND ANALYSIS

The QUOTA trial commenced on 1st April 2017 and consists of two stages: Stage 1 consisted of a series of three expert workshops which were conducted with the aim of designing the QUOTA research protocol. Stage 2 consists of the feasibility RCT.

Stage 1: Expert workshop summary

The research study measures and medication management protocol were designed through a series of three expert workshops held from April – July 2017. The workshops consisted of up to 21 multidisciplinary experts including: 4 patient and public involvement (PPI) members (parents of young people with ADHD; including co-author NB), 1 education expert, 2 representatives and clinical advisors from Qbtech, 1 health economics expert, 9 healthcare professionals (including consultant psychiatrists, paediatricians and nurse specialists incorporating co-authors CH, KS, JC, KSe), 2 academic team members (MG and CLH), and 2 representatives from National Institute for Health Research (NIHR) Health Technology Assessment (HTA) MindTech (JM and SB), who also bought additional PPI expertise.

Through group discussion the expert panel made decisions on: the role and frequency of the QbTest in the medication protocol, the selection and frequency of outcome measures, the design of the health economic resource use measures and clinician pro forma.

Stage 2: Feasibility randomised controlled trial

Trial design

The study is a parallel group, single-blind multi-centre feasibility RCT, which explores feasibility and acceptability of a QbTest medication management protocol, using quantitative, qualitative and health economic evaluations. The trial is registered with

For peer review only - http://bmjopen.bmj.com/site/about/guidelines.xhtml

www.clinicaltrials.gov (NCT03368573). The study flow is outlined in Figure 1. The trial consists of two arms:

1) Experimental arm (QbTest group)

In this arm, participants will receive standard treatment as usual plus three QbTests. The participant will complete a QbTest at baseline, prior to medication initiation (if a QbTest has not already been completed <12 week prior to medication initiation as part of their diagnosis), and again at follow-up 1 (2-4 weeks later), and follow-up 2 (8-10 weeks later).The clinician will utilise the QbTest scores to inform their clinical decision making regarding medication decisions (i.e., to inform titration, drug choice or treatment switch/termination).

2) Control arm (Treatment as usual)

In this arm, participants will receive standard treatment as usual. However, to provide some control over the possible increased clinical contact in the experimental arm, clinicians' are requested to make at least two contacts with the participant during the 12-week follow-up period. These contacts may take place over telephone.

<<INSERT FIGURE 1 HERE>>

The patients usual care team will be responsible for conducting the QbTest in clinic appointments. The QbTest will be only be conducted by trained QbTest clinicians. Although treatment as usual differs across sites/clinicians'/cases, it typically involves clinical interviews with the parents/carer/young person to ascertain improvement in symptoms, and sometimes collection of standardised outcome measures. The treatment-as-usual (TAU) care

For peer review only - http://bmjopen.bmj.com/site/about/guidelines.xhtml

practices will be recorded on a specifically created clinician completed pro forma (see measures section). There are no prohibited concomitant interventions. Given this study is assessing the feasibility of the protocol no measures will be taken to improve protocol adherence.

Setting

Child and Adolescent Mental Health Services (CAMHS) and Community Paediatric clinics across three different NHS Trusts in England are participating in the trial, including: Medway NHS Foundation Trust (KSe), United Lincolnshire Hospitals NHS Trust (JC), North East London NHS Foundation Trust (HV).

Recruitment and eligibility

Recruitment is scheduled to start 1st December 2017. Patients with a confirmed ADHD diagnosis and commencing stimulant medication for ADHD will be invited to participate in the research based on the following criteria:

Inclusion criteria

- Age 6-17-years-old (at the time of consent)
- Male or female
- Referred to CAMHS or Community Paediatric services
- Diagnosed with ADHD
- Clinician and family (parent/carer and young person/child) agreement to commence stimulant medication for ADHD symptoms
- Capable of providing written informed consent (over 16-years-old)
- Parental consent (under 16-years-old)

For peer review only - http://bmjopen.bmj.com/site/about/guidelines.xhtml

Exclusion criteria

- Unable to provide informed consent
- Severe learning disability (to be assessed by clinical judgment)
- Non-fluent English
- Not commencing stimulant medication (either not started on medication at all or started on a non-stimulant medication)

Eligibility will be determined by the treating clinician who has read and approved the protocol. Written information about the trial will be provided to families by their treating clinical team at the point where a decision to start stimulant medication has been agreed. There are four types of participant information sheets; one for parents/caregivers, one for young people aged 16-years and older, one for young people aged 12-15-years-old and one for children aged 6-11 years-old. The information sheets were developed with our PPI group. Clinicians will be encouraged to ask patients if they have any questions/queries before signing consent and will have sufficient knowledge of the research protocol to answer anticipated questions. Families may consent into the study at the appointment they first receive the information sheet, once they have had time to discuss the study and ask any questions with the clinician. The PPI group felt this would not put undue stress on families and was necessary to avoid any delays in medication initiation for those wishing to participate. Clinic invitations will be updated on a password protected database, recording numbers invited, numbers declined and reasons for decline. Each site will be informed of their monthly recruitment target required in order to meet the target sample size.

Measures

Blinded outcome assessors will be fully trained in all trial assessments and will be responsible for the delivery, monitoring, completion and data entry of all outcome measures.

For peer review only - http://bmjopen.bmj.com/site/about/guidelines.xhtml

Given this is a feasibility RCT, there is no specified primary outcome. The primary outcome for a future definitive RCT will be selected through our workshops (stage 1) and post-intervention interviews (stage 2) as being most clinically meaningful and important to our workshop experts, and also is shown to be acceptable for participants to complete in the feasibility RCT. The measures collected during this trial include:

- SNAP-IV[28]: The SNAP-IV is a short, 26-item questionnaire designed to assess ADHD symptoms. The SNAP-IV will be completed by parents/carers and teachers at baseline, follow-up 1 and follow-up 2. A 25% reduction in scores from baseline to follow-up 2 was identified in the workshops (Stage 1) as an appropriate potential primary outcome measure.
- Strengths and Difficulties Questionnaire (SDQ[29]): The SDQ is a brief, 25-item behavioural screening questionnaire which can be used as part of a clinical assessment for ADHD. The questionnaire also contains a brief impact supplement which assesses the burden and impact of symptoms[30]. The SDQ will be completed by parents/carers and teachers at baseline and follow-up 2.
- Clinical Global Impressions scale (CGI[31]): The CGI is a clinician completed measure designed to measure the clinician view of global functioning prior to, and after, treatment initiation. The questionnaire consists of two items, one measuring symptom severity and one measuring change since treatment. The CGI will be completed by the clinician at baseline and follow-up 2.
- Child Health Utility (CHU9D[32]: The CHU9D is a quality of life measure designed for the economic evaluation of interventions for young people. The CHU9D will be completed by parents/carers/young people at baseline, follow-up 1 and follow-up 2.

For peer review only - http://bmjopen.bmj.com/site/about/guidelines.xhtml

- QbTest: Q scores for attention, impulsivity and activity will be compared between the two groups. The QbTest is completed by the young person at baseline (if not conducted as part of the diagnostic assessment within 12-weeks of medication initiation), follow-up 1, and follow-up 2.
- Side effects scale[33]. A side effects scale will be completed by parents/carers/young people and teachers at follow-up 1 and follow-up 2 to check for any differences in side-effects between the two treatment groups.
- Medication adherence questionnaire: To ascertain that participants have been taking medication, they will be asked to complete a brief questionnaire created specifically for this study which asks how often they have taken their medication over the past 4-weeks. The questionnaire will be completed by parents/carers/young people at follow-up 1 and follow-up 2.
- Resource Use - Services for Health (RUSH) and Resource Use – Services in Education (RUSE): To collect relevant health economic information two tailored resource use tools will be used to measure the use of services used by the family and to ascertain indirect costs (such as time off work). More specifically, the RUSE measures the use of additionally education resources. The measures were based on the Client Service Receipt Inventory (CSRI [34], and refined for use in this study by our multidisciplinary group of members (including PPI, clinicians, and a health economic expert [MJ]) in our expert workshops (Stage 1). The measures will be completed by parents (RUSH) and teachers (RUSE) at follow-up 2.
- Clinical pro forma: As part of the expert workshops, a specifically created pro forma was designed for completion by clinicians after each consultation with the young person and/or family. The pro forma documents information about appointment

For peer review only - http://bmjopen.bmj.com/site/about/guidelines.xhtml

duration, diagnosis and changes to medication/treatment. The pro-forma can be provided by contacting the corresponding author.

The SNAP-IV[35], SDQ[36], CGI[37], CHU9D[38], and side-effects scale[33] have established reliability, validity and history of use in clinical and research settings. As this is a feasibility study there are no plans to promote retention and follow-up measure completion, but completion rates will be recorded to inform the future RCT.
Additionally, a sub-sample of 10-15 participants (parents/carers and/or young people) in the experimental arm will be interviewed about their experiences of the trial, including their opinion on randomisation; within this sub-sample, we will include participants who did not complete the trial, if possible, acknowledging that the views and experiences of non-completers may also provide useful insight into acceptability. A sub-sample of 10 in the control arm will be interviewed about their experiences of ADHD medication. The sub-sample will be chosen at random from each participating site, using a random number generator. All clinicians participating in the feasibility RCT will be interviewed, and asked to comment on any local factors that influenced delivery of the protocol at their site, providing early insight into factors that might influence delivery of the multi-site RCT and future implementation of the protocol into the NHS. All interviews will take place after their duration in the RCT has been completed to avoid any impact on outcome measures. Interviews will be digitally recorded, transcribed and analysed. The results of these interviews will be used to inform any refinement of the protocol to improve its acceptability before embarking on the definitive RCT.

Table 1 displays the study measures, the informant and the time point of completion. All measures will have a one-month window for completion, with the exception of the clinic pro

For peer review only - http://bmjopen.bmj.com/site/about/guidelines.xhtml

forma which must be just after the clinic appointment and the QbTest which will be completed within the specified time frame. . For participants who withdraw from the trial, the outcome measures already collected will be included in analysis, and no further outcome measures will be collected.

Table 1. Table of study measures

Measure (and informant)	Baseline	Follow-up 1 (2-4 weeks)	Follow-up 2 (8-10 weeks)
SNAP-IV (P&T)	x	x	x
SDQ (P&T)	x		x
CGI (C)	x		x
CHU9D (P&YP)	x	x	x
Medication adherence (P&YP)		x	x
Side effects (P&YP)		x	x
QbTest (YP)*	x	x	x
RUSH (P)			x
RUSE (T)			x
Pro forma (C)+	x	x	x
Sub-sample for interview (P, YP, C)			x

Note. *Experimental arm only. +Pro forma completed at every appointment. C = Clinician. P = Parent/carer. T = Teacher completed. YP = Young person. SDQ = Strengths and Difficulties Questionnaire. RUSH = Resource Use – Services for Heatlh. .RUSE = Resource Use - Services in Education. CHU9D = Child Health Utility 9DCGI = Clinical Global Impression scale.

Sample size and justification

The required sample is 60 participants, 30 per study arm. Participants will be families (parents/carers and children/young people) whose child/young person is about to commence stimulant medication for ADHD. These sample sizes are large enough to test the feasibility of the research procedures and to establish a mean and standard deviation on each outcome

For peer review only - http://bmjopen.bmj.com/site/about/guidelines.xhtml

measure (Hertzog; 2008). The study has an eight-month recruitment period, requiring 7.5 participants to be recruited into the study each month. Based on findings from the AQUA-Trial (which included the three sites used in this trial) this target is achievable. Recruitment rates and the final target will be used to inform the decision to proceed to a definitive RCT.

Randomisation and blinding

After obtaining informed consent, participants will be randomised on a 1:1 ratio into either the QbTest medication arm (experimental arm) or treatment as usual (control arm). Randomisation will take place via sealed opaque envelopes generated by our study statistician (BG). The sealed envelopes will be provided to the clinic sites and opened at the point of consent by the clinician.

All participants will undergo the same research measures, with the exception of the QbTest, which will only be in the experimental arm. Outcome assessors for all measures will be blind to which arm the participant is in. There are no anticipated events in which participant un-blinding would be necessary.

Data analysis plan

As a feasibility study, data analysis will be mainly descriptive, as recommended by Lancaster[39] and Lancaster, et al.[40]. All measures will be summarised by group across follow-up time with mean (SD) for normally distributed data, median (IQR) for skewed variable and frequency (percentage) for categorical measures. Together with site level intra-class correlation coefficient, treatment effects and 95% confidence interval will be derived using multi-level modelling. Recruitment rate and retention rate will also be calculated from the

For peer review only - http://bmjopen.bmj.com/site/about/guidelines.xhtml

data. This information will be used to inform the future definitive RCT design. All statistical analysis will be conducted using STATA15. No interim analysis is planned.

To inform the acceptability and feasibility of the study design the following indices will be recorded and analysed: (1) *acceptability of randomisation* - record of the number of patients who do not participate stating randomisation as the reason for non-participation, drop-out rates of randomisation, errors in randomisation per site; (2) *acceptability of study design* - record of the number of eligible participants at each site and the percentage that consent to take part in the study, number of withdrawals at each follow-up time point; (3) *acceptability of outcome measure*s - record of completion rates for outcome measures, percentage of data collected online, via telephone, or postal completion; (4) *acceptability/feasibility of the protocol* – record of non-adherence of healthcare professionals to the protocol. These reasons will be further explored in the qualitative interviews; (5) *feasibility of a future definitive RCT* – record an estimate of the hours per week spent conducting the RCT and estimate the number of researchers required and the time commitment for healthcare professionals in a future RCT.

The qualitative interviews will shed light on contextual and other factors that might affect implementation of QbTest (both as part of the trial, and within the broader processes of care) and will be used to refine the RCT design (and QbTest implementation more broadly) if appropriate. The qualitative interviews will be transcribed verbatim and analysed thematically following the guidelines of Braun and Clarke[41].The quantitative and qualitative findings will be used to determine the feasibility and acceptability of the medication protocol and research study design, and inform the decision to proceed to a fully-powered RCT.

For peer review only - http://bmjopen.bmj.com/site/about/guidelines.xhtml

Monitoring

Management and oversight

Recruitment and study progress will be overseen by our project management group (PMG), which includes all site principal investigators (JC, KSe, HV), a PPI representative (NB), the chief investigator (MG), the trial manager (CLH) and the study team (CH, SB, MJ, BG, KS). The PMG will meet every six-months, however, any severe slippages in recruitment or study milestones will be reported to the group immediately by the trial manager (CLH). Given this is a feasibility study, a data monitoring committee is not necessary.

Adverse events

All adverse events that occur will be assessed for seriousness, expectedness and causality. The chief investigator (MG) and the medical expert (CH), shall be informed immediately of any serious adverse events and shall determine seriousness and causality in conjunction with any treating medical practitioners. All treatment related serious adverse events will be recorded and reported to the REC. There are no anticipated adverse events arising from this study.

Audit

The Trial Coordinator, or a nominated designee of the Sponsor, shall carry out monitoring of trial data as an ongoing activity. A sample (10%) of case report forms (CRFs) will be checked on a regular basis for verification of all entries made. Where corrections are required these will carry a full audit trail and justification. Trial data and evidence of monitoring and systems audits will be made available for inspection by the Research Ethics Committee (REC) as required.

For peer review only - http://bmjopen.bmj.com/site/about/guidelines.xhtml

ETHICS AND DISSEMINATION

The study received ethical approval from West of Scotland REC 1 (17/WS/0209: protocol version 1.1, 7th November 2017). Health Research Authority (HRA) approvals have been granted from the three participating Trusts. The study is sponsored by Nottinghamshire Healthcare NHS Foundation Trust; neither the sponsor nor the funders (or Qbtech Ltd) will be involved in the analysis of study data or report writing. Only the research team will have access to the study data, which will be stored in secure locked files or password protected databases. Data will be available for inspection by the ethics committee upon request. Changes to the protocol will be communicated to the ethics committee and trial registries by the trial manager (CLH). The process for obtaining participant informed consent or assent and parent / guardian / teacher informed consent will be in accordance with the ethical guidance, and Good Clinical Practice (GCP). The investigator or their nominee and the participant or other legally authorised representative (such as the child's parent) shall both sign and date the informed consent forms (Appendix A & B) before the person can participate in the study. Where the young person is 16-years and over, written consent will be required from the young person and parent alike. Where the young person is under 16-years, written parental consent will be required, alongside the young person's written or verbal assent. Teachers will also be asked to sign a consent form (Appendix C), if teachers do not sign consent the participant is still eligible for the study but no teacher measures will be collected. Individual participant medical information obtained as a result of this study are considered confidential and disclosure to third parties is prohibited unless warranted by an adverse event. Participant confidentiality will be further ensured by utilising identification code numbers to correspond to treatment data in the computer files. No post-trial care is

For peer review only - http://bmjopen.bmj.com/site/about/guidelines.xhtml

required. The chief investigators and site principal investigators declare no financial or competing interests.

The findings from the trial will be used to inform the design, feasibility and acceptability of a future, fully-powered RCT. The findings will be published in peer-reviewed journals, presented at relevant conferences and disseminated to the public via lay summaries co-created with our PPI group. All outputs will be authored by the research team and will not involve professional writers. Access to the full protocol and statistical codes are available upon request to the corresponding author.

Acknowledgements: We would like to thank all members of our expert workshops who helped us develop this protocol, including: Dr. Maria Moldavsky, Dr. Carsten Vogt, Joe Kilgariff, Dr Margaret Murphy, Dr Rosemary Gradwell, Christine Jarvis, Anna Saunders, Zoe Coles and Helen Mellor. We would like to extend our thanks and appreciation to members of Qbtech: Tony Doyle, Charlotte Keizer, Hans Bostrom and Fredrik Ulberstad for their advice and ongoing support. Finally, we would like to thank Angela Summerfield for her continued support and help.

Contributors: MG is the Chief Investigator and takes final responsibility for study design, conduct and decision to submit for publication. The study design was conceived by MG, CLH (senior research fellow) and CH (co-investigator). CLH and MG wrote the protocol with approval from all authors. BG (statistician and co-investigator) designed and wrote the statistical analysis plan. MJ (health economist and co-investigator) advised on the health economic data collection and analysis. SB, KSa, KSe, CH, JM, JC, HV and NB (co-investigators) provided advice and critical input on the study design. NB (co-investigator) led on patient and public involvement. SB provided additional expertise on qualitative analysis.

For peer review only - http://bmjopen.bmj.com/site/about/guidelines.xhtml

KSa, KSe, HV, JC, CH provided additional clinical expertise. JM provided additional expertise on technology in health designs. All authors critically revised the manuscript for its important intellectual content. All authors read and approved the final manuscript.

Funding: This paper presents independent research funded by the National Institute for Health Research (NIHR) Research for patient benefit (RfPB), grant number PB-PG-1215-20026. The views expressed are those of the author(s) and not necessarily those of the NHS, the NIHR or the Department of Health. The study sponsor and funders have no role in study design, including; collection, management, analysis and interpretation of data; writing of the report; and the decision to submit the report for publication.

Trial Sponsor: Nottinghamshire Healthcare NHS Foundation Trust; Shirley Mitchell. Duncan Macmillan House, Porchester Road, Mapperley, Nottingham, UK, NG3 6AA Shirley.mitchell@nottshc.nhs.uk (Ref: Groom050917).

Competing interests: On behalf of all authors, the corresponding author declares no competing interests.

Ethical approval: This protocol (v1.1) was approved by West of Scotland REC 1 (REC reference 17/WS/0209) on 7th November 2017.

For peer review only

REFERENCES

1. NICE. Attention deficit hyperactivity disorder:diagnosis and managment of ADHD in children, young people and adults. *Clinical Guideline 72*. London: National Institute for Health and Clinical Excellence 2008.

For peer review only - http://bmjopen.bmj.com/site/about/guidelines.xhtml

2. Faraone SV, Biederman J, Spencer T, et al. Attention-deficit/hyperactivity disorder in adults: an overview. *Biol Psychiatry* 2000;48:9-20.

3. Wilens TE. Impact of ADHD and its treatment on substance abuse in adults. *Journal of Clinical Psychiatry* 2004;65:38-45.

4. D'Amico F, Knapp M, Beecham J, et al. Use of services and associated costs for young adults with childhood hyperactivity/conduct problems: 20-year follow-up. *BJPsych* 2014;204:441-47.

5. Schlander M. Impact of Attention-Deficit/Hyperactivity Disorder (ADHD) on prescription drug spending for children and adolescents: increasing relevance of health economic evidence. *Child Adolesc Psychiatry Ment Health* 2007;1(1):13.

6. King S, Griffin S, Hodges Z, et al. A systematic review and economic model of the effectiveness and cost-effectiveness of methylphenidate, dexamfetamine and atomoxetine for the treatment of attention deficit hyperactivity disorder in children and adolescents. *Health Technol Assess (Winchester, England)* 2006;10(23):iii-iv, xiii-146.

7. Hall CL, Taylor JA, Newell K, et al. The challenges of implementing ADHD clinical guidelines and research best evidence in routine clinical care settings: Delphi survey and mixed-methods study. *BJPsych Open* 2016;2:25-31.

8. England N. Implementing the five year forward view for mental health. *London: NHS England* 2016.

9. MTA Group. A 14-month randomized clinical trial of treatment strategies for attention-deficit/hyperactivity disorder. *Arch Gen Psychiatry* 1999;56(12):1073.

10. Simons L, Valentine AZ, Falconer CJ, et al. Developing mHealth remote monitoring technology for attention deficit hyperactivity disorder: a qualitative study eliciting user priorities and needs. *JMIR mHealth and uHealth* 2016;4(1).

For peer review only - http://bmjopen.bmj.com/site/about/guidelines.xhtml

11. Sonuga-Barke EJ, Sergeant JA, Nigg J, et al. Executive dysfunction and delay aversion in attention deficit hyperactivity disorder: nosologic and diagnostic implications. *Child Adolesc Psychiatr Clin N Am* 2008;17:367-84.

12. Ogundele MO, Ayyash HF, Banerjee S. Role of computerised continuous performance task tests in ADHD. *Prog Neurol Psychiatry* 2011;15:8-13.

13. Fernandez-Jaen A, Fernandez-Mayoralas DM, Pardos A, et al. Clinical and cognitive response to extended-release methylphenidate (Medikinet) in attention deficit/hyperactivity disorder: efficacy evaluation. *Advances in Therapy* 2009;26:1097-110.

14. Huang-Pollock CL, Karalunas SL, Tam H, et al. Evaluating vigilance deficits in ADHD: a meta-analysis of CPT performance. *J Abnorm Psychol* 2012;121:360-71.

15. Solanto MV, Etefia K, Marks DJ. The utility of self-report measures and the continuous performance test in the diagnosis of ADHD in adults. *CNS Spectrums* 2004;9:649-59.

16. Hall CL, Valentine AZ, Groom MJ, et al. The clinical utility of the continuous performance test and objective measures of activity for diagnosing and monitoring ADHD in children: a systematic review. *Eur Child Adolesc Psychiatry* 2016; 25:677-699.

17. Ulberstad F. *QbTest Technical Manual*. Stockholm, Sweden: Qbtech *AB* 2012

18. Vogt C, Shameli A. Assessments for attention-deficit hyperactivity disorder: Use of objective measurements. *The Psychiatrist* 2011;35:380-83.

19. Sharma A, Singh B. Evaluation of the role of Qb testing in attention deficit hyperactivity disorder. *Arc Dis Child* 2009;94(Suppl 1):A72.

20. Groom MJ, Young Z, Hall CL, et al. The incremental validity of a computerised assessment added to clinical rating scales to differentiate adult ADHD from autism spectrum disorder. *Psychiatry Research* 2016;243:168-73.

For peer review only - http://bmjopen.bmj.com/site/about/guidelines.xhtml

21. Hall CL, Selby K, Guo B, et al. Innovations in Practice: an objective measure of attention, impulsivity and activity reduces time to confirm attention deficit/hyperactivity disorder diagnosis in children–a completed audit cycle. *Child and Adolescent Mental Health* 2016;21:175-78.

22. Hall CL, Walker GM, Valentine AZ, et al. Protocol investigating the clinical utility of an objective measure of activity and attention (QbTest) on diagnostic and treatment decision-making in children and young people with ADHD—'Assessing QbTest Utility in ADHD'(AQUA): a randomised controlled trial. *BMJ Open* 2014;4(12):e006838.

23. Hall CL, Valentine AZ, Walker GM, et al. Study of user experience of an objective test (QbTest) to aid ADHD assessment and medication management: a multi-methods approach. *BMC Psychiatry* 2017;17(1):66.

24. Dam M, Kolmos K, Bilenberg N. Does Test Dose of Central Stimulant Influence Continuous Performance Test (CPT) and Activity in Boys with Attention Deficit Hyperactivity Disorder. *Clin Psychiatry* 2016;2(3) doi: 10.21767/2471-9854.100026

25. Wehmeier PM, Schacht A, Wolff C, et al. Neuropsychological outcomes across the day in children with attention-deficit/hyperactivity disorder treated with atomoxetine: results from a placebo-controlled study using a computer-based continuous performance test combined with an infra-red motion-tracking device. *J Child Adolesc Psychopharmacol* 2011;21:433-44.

26. Vogt C, Williams T. Early identification of stimulant treatment responders, partial responders and non□responders using objective measures in children and adolescents with hyperkinetic disorder. *Child and Adolescent Mental Health* 2011;16(3):144-49.

For peer review only - http://bmjopen.bmj.com/site/about/guidelines.xhtml

27. Bijlenga D, Jasperse M, Gehlhaar S, et al. Objective QbTest and subjective evaluation of stimulant treatment in adult attention deficit-hyperactivity disorder. *European Psychiatry* 2015;30:179-85.

28. Swanson JM, Sandman CA, Deutsch C, et al. Methylphenidate hydrochloride given with or before breakfast: I. Behavioral, cognitive, and electrophysiologic effects. *Pediatrics* 1983;72:49-55.

29. Goodman R. The Strengths and Difficulties Questionnaire: a research note. *J Child Psychol Psychiatry* 1997;38:581-86.

30. Goodman R. The extended version of the Strengths and Difficulties Questionnaire as a guide to child psychiatric caseness and consequent burden. *J Child Psychol Psychiatry* 1999;40:791-99.

31. Guy W. CGI clinical global impressions. *EC-DEU Assessment Manual for Psychopharmacology* 1976:76-338.

32. Stevens K. The Child Health Utility 9D (CHU9D). A new, paediatric, preference-based measure of health related quality of life. *PRO Newsletter* 2010;43:11-2.

33. Hill P, Taylor E. An auditable protocol for treating attention deficit/hyperactivity disorder. *Arch Dis Childhood* 2001;84:404-09.

34. Beecham J, Knapp M. *Costing Psychiatric Interventions*. In: Thornicroft G editior. Measuring Mental Health Needs. London: Gaskell, 2001.

35. Bussing R, Fernandez M, Harwood M, et al. Parent and teacher SNAP-IV ratings of attention deficit hyperactivity disorder symptoms: psychometric properties and normative ratings from a school district sample. *Assessment* 2008;15:317-28.

36. Goodman R. Psychometric properties of the strengths and difficulties questionnaire. *J Am Acad Child Adolesc Psychiatry* 2001;40:1337-45.

For peer review only

For peer review only - http://bmjopen.bmj.com/site/about/guidelines.xhtml

37. Busner J, Targum SD. The clinical global impressions scale: applying a research tool in clinical practice. *Psychiatry* 2007;4(7):28.

38. Furber G, Segal L. The validity of the Child Health Utility instrument (CHU9D) as a routine outcome measure for use in child and adolescent mental health services. *Health Qual Life Outcomes* 2015;13(1):22.

39. Lancaster GA. Pilot and feasibility studies come of age! *Pilot Feasibility Stud* 2015;1(1):1.

40. Lancaster GA, Dodd S, Williamson PR. Design and analysis of pilot studies: recommendations for good practice. *J Eval Clin Pract* 2004;10(2):307-12.

41. Braun V, Clarke V. Using thematic analysis in psychology. *Qual Res Psychol* 2006;3:77-101.

FIGURE CAPTION:

Figure 1: Participant Flow Diagram

For peer review only

For peer review only - http://bmjopen.bmj.com/site/about/guidelines.xhtml

Figure 1

FIGURE 1: PARTICIPANT FLOW DIAGRAM

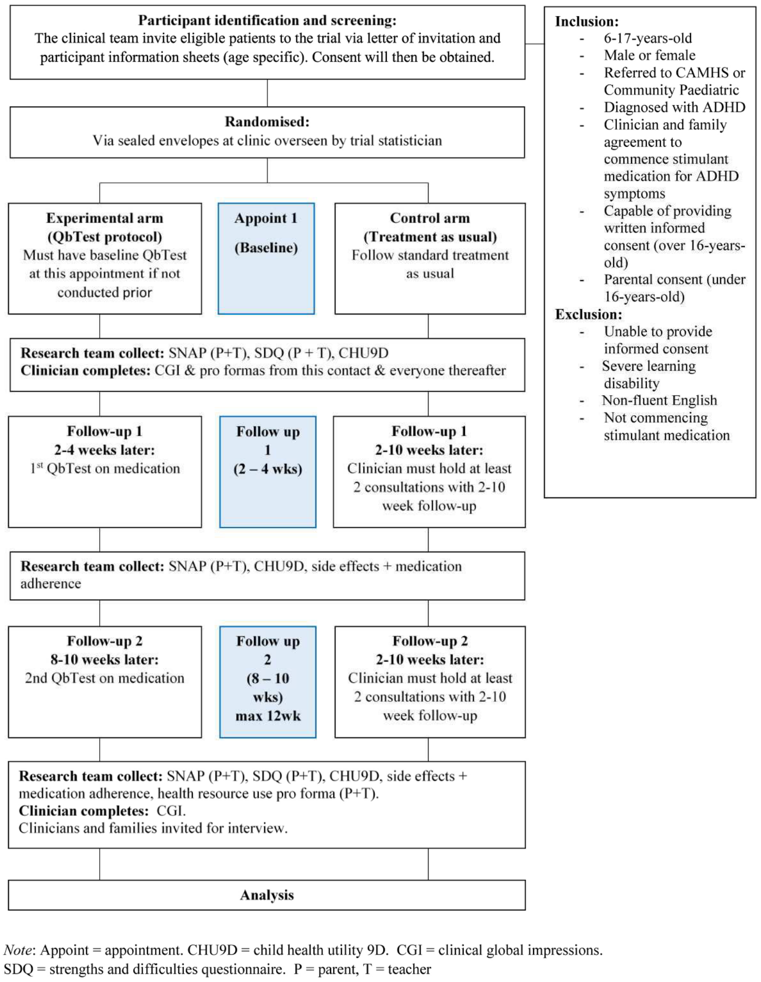

Figure 1: Participant Flow Diagram

170x249mm (300 x 300 DPI)

Appendix A INSERT TRUST LOGO
V1.0: 30.08.2017

Study ID: IRAS 219538 **Participant Identification Number:**

Name of participant (child):

CONSENT FORM

Title of Project: QbTest Utility for Optimising Treatment in ADHD (QUOTA)

Name of Researcher: **CI: Dr Maddie Groom.**

Please initial all boxes

1. I confirm that I have read and understand the information sheet dated 10th Oct 2017 version 1.1 for the above study. I have had the opportunity to consider the information, ask questions and have had these answered satisfactorily. ☐

2. I understand that my/my child's participation is voluntary and that I am free to withdraw at any time without giving any reason, without my/my child's medical care or legal rights being affected. ☐

3. I understand that relevant sections of my child's medical notes and data collected during the study, may be looked at by individuals from the University of Nottingham, from regulatory authorities or from the NHS Trust, where it is relevant to my taking part in this research. I give permission for these individuals to have access to my child's records. ☐

4. I agree to my child's teacher and head teacher to be contacted and be asked to complete questionnaires about their behaviour at school and provide a Provision Map if available. ☐

5. I understand if I/my child takes part in any research interviews, they will be recorded and that anoymous direct quotes from the interviews may be used in study reports. ☐

6. I agree to take part in the above study. ☐

________________________	________________________	________________________
Name of parent/care giver	Date	Signature

________________________	________________________	________________________
Name of person taking consent	Date	Signature

OPTIONAL: for child assent

________________________	________________________	________________________
Name of child	Date	Signature

QbTest Utility for Optimising Treatment in ADHD. Version 1.0, consent parent, 30-AUG-2017, IRAS 219538

For peer review only

Appendix B

INSERT TRUST LOGO

V1.0: 30.08.2017

Study ID: IRAS 219538 **Participant Identification Number:**

Name of participant (young person):

CONSENT FORM

Title of Project: QbTest Utility for Optimising Treatment in ADHD (QUOTA)

Name of Researcher: **CI: Dr Maddie Groom.**

Please initial all boxes

1. I confirm that I have read and understand the information sheet dated 10th Oct 2017 version 1.1 for the above study. I have had the opportunity to consider the information, ask questions and have had these answered satisfactorily. ☐

2. I understand that my participation is voluntary and that I am free to withdraw at any time without giving any reason, without my medical care or legal rights being affected. ☐

3. I understand that relevant sections of my medical notes and data collected during the study, may be looked at by individuals from the University of Nottingham, from regulatory authorities or from the NHS Trust, where it is relevant to my taking part in this research. I give permission for these individuals to have access to my records. ☐

4. I agree to my teacher and head teacher to be contacted and be asked to complete questionnaires about my behaviour at school and provide a Provision Map if avalaible. ☐

5. I understand if I take part in any research interviews, they will be recorded and that anoymous direct quotes from the interviews may be used in study reports. ☐

6. I agree to take part in the above study. ☐

Name of participant	Date	Signature
Name of person taking consent	Date	Signature

QbTest Utility for Optimising Treatment in ADHD. Version 1.0, consent 16plus, 30-AUG-2017, IRAS 219538

For peer review only

Appendix C INSERT TRUST LOGO

V1.0: 10.10.2017

Study ID: IRAS 219538 **Participant Identification Number:**

Name of participant (child):

CONSENT FORM

Title of Project: QbTest Utility for Optimising Treatment in ADHD (QUOTA)

Name of Researcher: **CI: Dr Maddie Groom.**

Please initial all boxes

1. I confirm that I have read and understand the information sheet dated 10th Oct 2017 version 1.1 for the above study. I have had the opportunity to consider the information, ask questions (by email or phone) and have had these answered satisfactorily (if appropriate). ☐

2. I understand that my participation is voluntary and that I am free to withdraw at any time without giving any reason, without the child's medical care or legal rights being affected. ☐

3. I understand that all data will be kept in accordance with the Data Protection Act (1998) and that no material which could identify individual children, teachers or the school will be used in any reports of this project. ☐

4. I agree to complete questionnaires about the child's behaviour at school and provide a Provision Map if available. ☐

5. I agree to take part in the above study. ☐

Your name (PLEAST PRINT)	Date	Signature

Your role e.g. class teacher, form teacher, SENCo

To be completed by the research team:

Name of person taking consent	Date	Signature

For peer review only - http://bmjopen.bmj.com/site/about/guidelines.xhtml

For peer review only

STANDARD PROTOCOL ITEMS: RECOMMENDATIONS FOR INTERVENTIONAL TRIALS

SPIRIT 2013 Checklist: Recommended items to address in a clinical trial protocol and related documents*

Section/item	Item No	Description	Addressed on page number
**Administrative information**			
Title	1	Descriptive title identifying the study design, population, interventions, and, if applicable, trial acronym	______1_______
Trial registration	2a	Trial identifier and registry name. If not yet registered, name of intended registry	______3______
	2b	All items from the World Health Organization Trial Registration Data Set	_____NA______
Protocol version	3	Date and version identifier	_____18_____
Funding	4	Sources and types of financial, material, and other support	_____20_____
Roles and responsibilities	5a	Names, affiliations, and roles of protocol contributors	_____1______
	5b	Name and contact information for the trial sponsor	_____20_______
	5c	Role of study sponsor and funders, if any, in study design; collection, management, analysis, and interpretation of data; writing of the report; and the decision to submit the report for publication, including whether they will have ultimate authority over any of these activities	_____20______
	5d	Composition, roles, and responsibilities of the coordinating centre, steering committee, endpoint adjudication committee, data management team, and other individuals or groups overseeing the trial, if applicable (see Item 21a for data monitoring committee)	_____18____

For peer review only

For peer review only - http://bmjopen.bmj.com/site/about/guidelines.xhtml

**Introduction**			
Background and rationale	6a	Description of research question and justification for undertaking the trial, including summary of relevant studies (published and unpublished) examining benefits and harms for each intervention	_____3-7_____
	6b	Explanation for choice of comparators	_____7_____
Objectives	7	Specific objectives or hypotheses	_____7______
Trial design	8	Description of trial design including type of trial (eg, parallel group, crossover, factorial, single group), allocation ratio, and framework (eg, superiority, equivalence, noninferiority, exploratory)	_____7________
**Methods: Participants, interventions, and outcomes**			
Study setting	9	Description of study settings (eg, community clinic, academic hospital) and list of countries where data will be collected. Reference to where list of study sites can be obtained	_____10______
Eligibility criteria	10	Inclusion and exclusion criteria for participants. If applicable, eligibility criteria for study centres and individuals who will perform the interventions (eg, surgeons, psychotherapists)	_____10-11____
Interventions	11a	Interventions for each group with sufficient detail to allow replication, including how and when they will be administered	___9 & fig 1____
	11b	Criteria for discontinuing or modifying allocated interventions for a given trial participant (eg, drug dose change in response to harms, participant request, or improving/worsening disease)	_____NA_____
	11c	Strategies to improve adherence to intervention protocols, and any procedures for monitoring adherence (eg, drug tablet return, laboratory tests)	_____10____
	11d	Relevant concomitant care and interventions that are permitted or prohibited during the trial	_____10____
Outcomes	12	Primary, secondary, and other outcomes, including the specific measurement variable (eg, systolic blood pressure), analysis metric (eg, change from baseline, final value, time to event), method of aggregation (eg, median, proportion), and time point for each outcome. Explanation of the clinical relevance of chosen efficacy and harm outcomes is strongly recommended	___11-15______
Participant timeline	13	Time schedule of enrolment, interventions (including any run-ins and washouts), assessments, and visits for participants. A schematic diagram is highly recommended (see Figure)	9, Table 1, fig 1

For peer review only - http://bmjopen.bmj.com/site/about/guidelines.xhtml

Sample size	14	Estimated number of participants needed to achieve study objectives and how it was determined, including clinical and statistical assumptions supporting any sample size calculations	___15-16______
Recruitment	15	Strategies for achieving adequate participant enrolment to reach target sample size	____11______
**Methods: Assignment of interventions (for controlled trials)**			
Allocation:			
Sequence generation	16a	Method of generating the allocation sequence (eg, computer-generated random numbers), and list of any factors for stratification. To reduce predictability of a random sequence, details of any planned restriction (eg, blocking) should be provided in a separate document that is unavailable to those who enrol participants or assign interventions	____16_______
Allocation concealment mechanism	16b	Mechanism of implementing the allocation sequence (eg, central telephone; sequentially numbered, opaque, sealed envelopes), describing any steps to conceal the sequence until interventions are assigned	____16______
Implementation	16c	Who will generate the allocation sequence, who will enrol participants, and who will assign participants to interventions	____16______
Blinding (masking)	17a	Who will be blinded after assignment to interventions (eg, trial participants, care providers, outcome assessors, data analysts), and how	_____16_____
	17b	If blinded, circumstances under which unblinding is permissible, and procedure for revealing a participant's allocated intervention during the trial	_____16______
**Methods: Data collection, management, and analysis**			
Data collection methods	18a	Plans for assessment and collection of outcome, baseline, and other trial data, including any related processes to promote data quality (eg, duplicate measurements, training of assessors) and a description of study instruments (eg, questionnaires, laboratory tests) along with their reliability and validity, if known. Reference to where data collection forms can be found, if not in the protocol	11-15 & Table 1
	18b	Plans to promote participant retention and complete follow-up, including list of any outcome data to be collected for participants who discontinue or deviate from intervention protocols	____14______

For peer review only - http://bmjopen.bmj.com/site/about/guidelines.xhtml

Data management	19	Plans for data entry, coding, security, and storage, including any related processes to promote data quality (eg, double data entry; range checks for data values). Reference to where details of data management procedures can be found, if not in the protocol	_____ 18,19___
Statistical methods	20a	Statistical methods for analysing primary and secondary outcomes. Reference to where other details of the statistical analysis plan can be found, if not in the protocol	_____16,17_____
	20b	Methods for any additional analyses (eg, subgroup and adjusted analyses)	_____16,17_____
	20c	Definition of analysis population relating to protocol non-adherence (eg, as randomised analysis), and any statistical methods to handle missing data (eg, multiple imputation)	______NA________
**Methods: Monitoring**			
Data monitoring	21a	Composition of data monitoring committee (DMC); summary of its role and reporting structure; statement of whether it is independent from the sponsor and competing interests; and reference to where further details about its charter can be found, if not in the protocol. Alternatively, an explanation of why a DMC is not needed	_____17-18_______
	21b	Description of any interim analyses and stopping guidelines, including who will have access to these interim results and make the final decision to terminate the trial	______17_______
Harms	22	Plans for collecting, assessing, reporting, and managing solicited and spontaneously reported adverse events and other unintended effects of trial interventions or trial conduct	_____18_______
Auditing	23	Frequency and procedures for auditing trial conduct, if any, and whether the process will be independent from investigators and the sponsor	_____18_______
**Ethics and dissemination**			
Research ethics approval	24	Plans for seeking research ethics committee/institutional review board (REC/IRB) approval	_____18-19_____
Protocol amendments	25	Plans for communicating important protocol modifications (eg, changes to eligibility criteria, outcomes, analyses) to relevant parties (eg, investigators, REC/IRBs, trial participants, trial registries, journals, regulators)	_____19________

For peer review only - http://bmjopen.bmj.com/site/about/guidelines.xhtml

Consent or assent	26a	Who will obtain informed consent or assent from potential trial participants or authorised surrogates, and how (see Item 32)	___19_______
	26b	Additional consent provisions for collection and use of participant data and biological specimens in ancillary studies, if applicable	___NA______
Confidentiality	27	How personal information about potential and enrolled participants will be collected, shared, and maintained in order to protect confidentiality before, during, and after the trial	___19_______
Declaration of interests	28	Financial and other competing interests for principal investigators for the overall trial and each study site	__19 & 20____
Access to data	29	Statement of who will have access to the final trial dataset, and disclosure of contractual agreements that limit such access for investigators	___19_______
Ancillary and post-trial care	30	Provisions, if any, for ancillary and post-trial care, and for compensation to those who suffer harm from trial participation	___19_______
Dissemination policy	31a	Plans for investigators and sponsor to communicate trial results to participants, healthcare professionals, the public, and other relevant groups (eg, via publication, reporting in results databases, or other data sharing arrangements), including any publication restrictions	___19_______
	31b	Authorship eligibility guidelines and any intended use of professional writers	___19_______
	31c	Plans, if any, for granting public access to the full protocol, participant-level dataset, and statistical code	___19_______
**Appendices**			
Informed consent materials	32	Model consent form and other related documentation given to participants and authorised surrogates	Appendix A&B
Biological specimens	33	Plans for collection, laboratory evaluation, and storage of biological specimens for genetic or molecular analysis in the current trial and for future use in ancillary studies, if applicable	___NA_______

*It is strongly recommended that this checklist be read in conjunction with the SPIRIT 2013 Explanation & Elaboration for important clarification on the items. Amendments to the protocol should be tracked and dated. The SPIRIT checklist is copyrighted by the SPIRIT Group under the Creative Commons "Attribution-NonCommercial-NoDerivs 3.0 Unported" license.

For peer review only - http://bmjopen.bmj.com/site/about/guidelines.xhtml
