# Supplementary material for: Protocol investigating the clinical utility of an objective measure of attention, impulsivity and activity (QbTest) for optimising medication management in children and young people with ADHD ‘QbTest Utility for Optimising Treatment in ADHD’ (QUOTA): a feasibility randomised controlled trial
Source: BMJ Open. 2018 Feb 15;8(2):e021104. doi: 10.1136/bmjopen-2017-021104 (PMC5829926; doi:10.1136/bmjopen-2017-021104)
Supplement: Reviewer comments [file bmjopen-2017-021104.reviewer_comments.pdf]

PEER REVIEW HISTORY

BMJ Open publishes all reviews undertaken for accepted manuscripts. Reviewers are asked to complete a checklist review form (**http://bmjopen.bmj.com/site/about/resources/checklist.pdf**) and are provided with free text boxes to elaborate on their assessment. These free text comments are reproduced below.

ARTICLE DETAILS

**TITLE (PROVISIONAL)**	Protocol investigating the clinical utility of an objective measure of attention, impulsivity and activity (QbTest) for optimising medication management in children and young people with ADHD 'QbTest Utility for Optimising Treatment in ADHD' (QUOTA): a feasibility randomised controlled trial
**AUTHORS**	Hall, Charlotte; James, Marilyn; Brown, Sue; Martin, Jen; Brown, Nikki; Selby, Kim; Clarke, Julie; Vijayan, Hena; Guo, Boliang; Sayal, Kapil; Hollis, Chris; Groom, Maddie

This article was not externally reviewed at BMJ Open. Protocols that have been independently assessed prior to submission to BMJ Open are usually fast-tracked to publication on the grounds that further substantial changes will not be possible. This independent assessment will usually be external, independent review for both a substantial grant award from a non-commercial or government funder and ethics approval.
